# Evaluation of the Impact of Meibomian Gland Dysfunction Using a Novel Patient-Reported Outcome Instrument

**DOI:** 10.1089/jop.2023.0080

**Published:** 2024-02-09

**Authors:** Layla Ajouz, Joelle Hallak, Rupali Naik, Ashley Nguyen, Cathy Zhao, Michael R. Robinson, Kelly K. Nichols

**Affiliations:** ^1^ Allergan (an AbbVie company), Irvine, California, USA.; ^2^ Noesis Healthcare Technologies, Redwood City, California, USA.; ^3^ School of Optometry, University of Alabama at Birmingham, Birmingham, Alabama, USA.

**Keywords:** meibomian gland dysfunction, quality of life, patient-reported outcome measures, Meibomian Gland Dysfunction Impact Questionnaire

## Abstract

**Purpose:**

This study was intended to characterize the impact of meibomian gland dysfunction (MGD) on patients' quality of life.

**Methods:**

In this prospective, multicenter, noninterventional clinical study (NCT01979887), eligible individuals (age ≥40 years; absence of uncontrolled ocular/systemic disease) were categorized, based on composite grading of ocular symptoms, Schirmer score, and meibum quality, into (1) non-MGD, (2) mild/moderate MGD, or (3) severe MGD cohorts. The MGD Impact Questionnaire (MGD IQ), a 10-item patient-reported outcome measure, was self-administered at clinic visit on day 1, and readministered on day 22 to assess intervisit agreement regarding MGD IQ responses.

**Results:**

In total, 75 subjects were assigned to the study cohorts (25 per cohort). Across cohorts, MGD IQ item scores rose incrementally with increasing MGD severity. The severe MGD cohort experienced greater difficulty with reading and performance of leisure activities, greater time on eye care, and greater bother with eye care and eye appearance than the mild/moderate MGD cohort (all *P* < 0.05). Compared with the non-MGD cohort, the mild/moderate MGD cohort had greater difficulty working on computer, whereas the severe MGD cohort had greater difficulty reading, driving, and performing leisure activities, more frequent difficulty with outdoor activities, more time on eye care, and greater bother with eye care (all *P* < 0.05). Intervisit agreement between MGD IQ responses was fair to moderate (weighted kappa statistic 0.33‒0.58).

**Conclusions:**

Vision-related activities are negatively impacted by increasing severity of MGD. The MGD IQ instrument can help characterize disease severity and amplify the patient's voice in patient-centric clinical research. ClinicalTrials.gov NCT01979887.

## Introduction

The meibomian glands, located on the posterior margins of the eyelids, secrete lipids (meibum) onto the ocular surface to form the outermost layer of the tear film, thereby providing lubrication and protection against evaporation.^1^ Meibomian gland dysfunction (MGD) is a chronic, diffuse abnormality of the meibomian glands, commonly characterized by terminal duct obstruction, and/or qualitative/quantitative changes in meibum secretion, which is associated with alterations in eyelid morphology and symptoms of ocular irritation, inflammation, and dry eye.^2^ MGD is thought to be caused primarily by terminal duct obstruction secondary to hyperkeratinization of the ductal epithelium, leading to meibum stasis and meibomian gland atrophy (“dropout”).^3,4^

MGD is a major cause of evaporative dry eye disease (DED), with loss of meibum quantity/quality resulting in tear film instability, ocular surface evaporation, tear hyperosmolarity, and ocular surface inflammation.^5,6^ Key influences in terminal duct obstruction may include aging, hormonal disturbances, contact lens wear, and refractive surgery.^6,7^ Symptoms of MGD comprise ocular irritation, burning, itching, soreness, red eyes, impaired or fluctuating vision, and ocular fatigue.^2,8^ Ocular signs include eyelid thickening, marginal telangiectasia, erythema, vascular engorgement, hyperkeratinization, and irregularity or notching of the eyelid margins.^2,8^

In addition to assessing signs and symptoms, gaining the patient's perspective on how MGD impacts their daily lives is important when evaluating therapeutic risks and benefits. Patient-reported outcome (PRO) measures capture aspects of disease burden and treatment benefit as experienced by the patient. To this end, the MGD Impact Questionnaire (MGD IQ) has been developed in accordance with the standards described in the U.S. Food and Drug Administration's (FDA's) PRO Guidance document,^9^ specifically for use in the MGD patient population. We report here PRO findings obtained on administration of the MGD IQ instrument in the setting of a clinical study conducted in subjects with and without MGD.

## Methods

This prospective, multicenter, noninterventional exploratory study (ClinicalTrials.gov identifier: NCT01979887), conducted at sites in the United States (2) and the European Union (1) between November 2013 and July 2014, was undertaken to evaluate the correlation between signs and symptoms of MGD, and the quantitative relationship between MGD severity and the patient-reported health impact of MGD (as expressed through MGD IQ item scores). The study protocol was approved by an institutional review board or ethics committee at each site, and written informed consent was obtained from all enrolled subjects before initiation of any study procedures. The study was conducted in accordance with the International Conference for Harmonization guidelines, applicable regulations, and the principles of the Declaration of Helsinki.

The study investigators at each site [Sall Medical Research Center, Artesia, CA (*n* = 1), University of Houston, TX (*n* = 2), and Ocular Technology Group, London, United Kingdom (*n* = 1)] were licensed optometrists or board-certified ophthalmologists with expertise in evaluating signs and symptoms of dry eye, and extensive experience in conducting clinical trials in ocular surface disease. Additionally, protocol-specific investigator training was performed by the sponsor before the commencement of the study.

### Cohort selection

The study population consisted of subjects with and without MGD. For study inclusion, subjects were required to be ≥40 years of age at the time of screening. Excluded from the study were subjects who, on screening for eligibility, had (1) uncontrolled ocular (apart from MGD) or systemic disease; (2) corneal or eyelid abnormalities (other than those caused by MGD); (3) current ocular infection, or nonkeratoconjunctivitis sicca, ocular surface inflammation, or intraocular inflammation within the previous 3 months; (4) a history of herpes keratitis; (5) corneal refractive surgery within the previous 2 years; (6) a history of punctal cautery at any time, or punctal plug insertion/removal within the previous 2 months, or anticipated use of either procedure during the study; (7) clinically significant anterior blepharitis, meibomitis or ocular rosacea within the previous 30 days; or (8) lid heating therapy, therapeutic gland expression, or meibomian gland probing within the previous 12 months or anticipated use of such procedures during the study.

On the day of study enrollment (day 1), subjects who were found to have used nonpreserved artificial tears within the past 6 h, worn eye makeup within the past 8 h, performed lid hygiene within the past 48 h, or worn a contact lens or used topical/systemic antibiotics, systemic antihistamines, calcineurin inhibitors, preserved artificial tears, or eyelash growth-stimulating products in the preceding 30 days were required to undergo a corresponding washout interval before commencing the study.

Based on assessments of ocular signs and symptoms performed on study enrollment (day 1), study subjects were assigned to the following cohorts: subjects without MGD (“non-MGD,” cohort 1), subjects with mild/moderate MGD (cohort 2), and subjects with severe MGD (cohort 3). Ocular symptoms (blurred vision, eye burning, eye dryness, eye pain, light sensitivity, eye itching, eye foreign body sensation, and overall ocular discomfort) were assessed using the Subject Ocular Symptom Questionnaire (SOSQ), and average symptom scores over the past 7 days were graded on a 0‒4 scale (0 = none, 4 = very severe). Evaluations of ocular signs comprised meibum quality score for each of the 6 central meibomian glands of the lower eyelid, Maximum Meibum Quality Score (MMQS), defined as the maximum score among expressible glands (graded as 0, 1, 2, or 3), as assessed by the investigator, and Schirmer tear test without anesthesia, for which a score of ≥7 mm/5 min (to exclude aqueous-deficient dry eye) was required for assignment to a cohort.

A composite grading scale based on SOSQ (Sum of 2 Worst Symptoms), Schirmer tear test, and MMQS criteria (Table 1), was used to categorize subjects as either non-MGD, mild/moderate MGD, or severe MGD. To be assigned to a cohort, at least 1 eye was required to meet the specified criteria for cohort categorization, and this eye (or the right eye if both eyes met the cohort qualifications) was designated as the study eye. The intention was to enroll 75 subjects (25 subjects per study cohort) who satisfied the cohort qualifications.

**Table 1.** Cohort Categorization Criteria

Cohort	MGD severity	Required investigator-graded MMQS*^a,b,c^*	Schirmer tear test (without anesthesia)*^b^*	Sum of scores of 2 worst symptoms on Ocular Symptom Questionnaire
1	Non-MGD	0 or 1	≥7 mm/5 min	0 to 4 with neither symptom scored >2
2	Mild/moderate	2	≥7 mm/5 min	
3	Severe	3	≥7 mm/5 min	≥4

^a^ Based on grading of 6 central glands in the lower eyelid. Meibum quality of each gland was graded on a scale of 0 = clear excreta or clear with small particles (normal viscosity); 1 = opaque excreta with normal viscosity; 2 = opaque excreta with increased viscosity (gel-like); 3 = secretions retain shape, or secretions do not completely express but a toothpaste-like substance can be seen at the opening of the orifice; and NE = nonexpressible (nothing at orifice or metaplastic).

^b^ MMQS and Schirmer tear test criteria must be met in the same eye (study eye).

^c^ None of the 6 central glands being graded may have a meibum quality score greater than the specified value.

MGD, meibomian gland dysfunction; MMQS, maximum meibum quality score among the 6 central glands graded in the lower eyelid.

### Study assessments and outcome measures

The study included 3 scheduled clinic visits: screening (up to day minus 90), study enrollment (day 1 ± 3 days), and study exit (day 22 ± 3 days). During the interval between enrollment and exit visits, study participants maintained their usual daily activities but were required to avoid use of specified medications (including topical ocular medications and artificial tears, and systemic antibiotics, retinoids, anti-inflammatory agents, and antihistamines), contact lens wear, and performance of punctal procedures (plug insertion/removal, and punctal cautery). At the screening visit, study eligibility was evaluated and baseline data (demographics, vital signs, medical and ophthalmic history, medication history, and concomitant medications) were collected. On completion of the study enrollment visit, subjects who satisfied the cohort qualifications additionally attended the study exit visit to ascertain the degree of concordance between outcome measurements obtained at the 2 time points.

Study assessments conducted at day 1 and 22 included the following measures:

#### Ocular symptoms

Ocular symptoms (blurred vision, burning, dryness, pain, light sensitivity, itching, foreign body sensation, and overall ocular discomfort) were evaluated for severity on a 0–4 scale (0 = none, 4 = severe) with the SOSQ, an instrument created for this study.

#### Meibomian gland function

The secretion quality of the central meibomian glands of the lower eyelid was evaluated using a Meibomian Gland Evaluator™ (TearScience). The MMQS for the secretion quality was graded using Mathers' 4-point “opacity‒viscosity” scale, ranging from 0 (clear excreta, or clear with small particles; normal viscosity) to 3 (secretions retain shape, or have toothpaste-like consistency).^10^

#### Tear production

Schirmer's test without anesthesia.

#### Patient-reported health outcomes

The impact of MGD symptoms was assessed with the MGD IQ. The MGD IQ is a 10-item PRO measure that captures those aspects of patients' daily lives that are most adversely affected by MGD (Table 2).

**Table 2.** Concepts Represented in the Meibomian Gland Dysfunction Impact Questionnaire

MGD IQ items	Item scoring
Item 1 Difficulty working on the computer	0 (“No difficulty”)‒5 (“Very difficult”)
Item 2 Difficulty reading	0 (“No difficulty”)‒5 (“Very difficult”)
Item 3 Difficulty performing leisure activities	0 (“No difficulty”)‒5 (“Very difficult”)
Item 4 Difficulty performing social activities	0 (“No difficulty”)‒5 (“Very difficult”)
Item 5 Difficulty driving	0 (“No difficulty”)‒5 (“Very difficult”)
Item 6 Difficulty performing outdoor activities	0 (“No difficulty”)‒5 (“Very difficult”)
Item 7 Frequency of difficulty with outdoor activities	0 (“Never”)‒5 (“Very frequent”)
Item 8 Time spent caring for eyes	0 (“None”)‒4 (“Very much”)
Item 9 Bother with time spent caring for eyes	0 (“Not bothered”)‒4 (“Very bothered”)
Item 10 Bother with appearance of eyes	0 (“Not bothered”)‒4 (“Very bothered”)

MGD IQ, MGD Impact Questionnaire.

The questionnaire includes assessments of difficulty encountered by patients in performing activities such as working on the computer, reading, driving, and participating in social, leisure, and outdoor activities, as well as assessments of the time and bother experienced by patients in looking after their eyes, and concern about the physical appearance of their eyes. Patients completing the questionnaire are instructed to base their responses on their recall of experiences over the past 7 days. Individual items 1‒7 (difficulty working on the computer, reading, driving, and performing social, leisure, and outdoor activities; and frequency of difficulty with outdoor activities) are scored on a 6-point scale, ranging from 0 (“No difficulty/Never”) to 5 (“Very much difficulty/Very frequent”), while items 8‒10 (time spent on eye care, bother with eye care, and bother with appearance of eyes) are scored on a 5-point scale ranging from 0 (“None/Not bothered”) to 4 (“Very much time/Very bothered”) (Table 2).

#### Visual acuity and refractive error

Best-corrected visual acuity (BCVA) was measured at 3-m distance, using manifest refraction, with a logMAR chart. BCVA was recorded separately for the right and left eye in the Snellen equivalent. Lens powers (diopters) required for correction of myopia/hyperopia (sphere) and astigmatism (cylinder) were recorded for each eye. Spherical equivalent (SE), an estimate of refractive error, was derived using the formula SE = sphere + (0.5 × cylinder) and used to classify eyes as either emmetropes (SE −0.5 to +0.5D), myopes (SE < −0.5D), or hyperopes (SE > +0.5).^11^

### Statistical analyses

Pairwise intercohort comparisons (mild/moderate MGD vs. non-MGD, severe MGD vs. non-MGD, severe MGD vs. mild/moderate MGD) of MGD IQ mean scores at days 1 and 22 were conducted using the Cochran–Mantel–Haenszel method with modified ridit (“*r*elative to an *id*entified d*i*s*t*ribution”) scores (a stratified Wilcoxon rank sum test), stratified by study site. All statistical comparisons were made at the *α* = 0.05 level without adjustment for multiple testing in this exploratory analysis. Intervisit (day 1 vs. day 22) agreement between MGD IQ responses was expressed as the proportion of subjects with the same item score at both visits, and as a weighted kappa statistic.

## Results

### Study population

Of a total of 177 subjects who were screened for study participation, 129 satisfied the eligibility criteria and were enrolled into the study. On the basis of ocular symptoms, MMQS, and Schirmer test scores obtained at the study enrollment (day 1) visit, 75 subjects who qualified for cohort placement were assigned to the 3 study cohorts (25 participants per cohort) and were requested to attend the study exit (day 22) visit. All but 2 subjects placed into study cohorts completed the study as planned: 1 subject in the mild/moderate MGD cohort discontinued due to a nonocular adverse event and 1 subject in the severe MGD cohort was lost to follow-up. The study population was of mean (standard deviation) age 55.4 (9.6) years, predominantly female [64.3% (83/129)], and generally either Black [42.6% (55/129)], Caucasian [37.2% (48/129)], or Hispanic [14.0% (18/149)] (Table 3).

**Table 3.** Demographic and Clinical Characteristics of the Study Population at Study Entry

	Cohort 1, non-MGD, (*n* = 25)	Cohort 2, mild/moderate MGD, (*n* = 25)	Cohort 3, severe MGD, (*n* = 25)
Age, years			
Mean (range)	52.0 (40–74)	52.8 (43–63)	58.8 (41–89)
Gender female (%)	64.0	64.0	72.0
Race (%)			
Caucasian	52.0	24.0	16.0
Black	28.0	60.0	44.0
Hispanic	4.0	8.0	32.0
Asian	4.0	4.0	8.0
Other	12.0	4.0	0
Ophthalmic history (%)^a^			
Overall	28.0	40.0	72.0
Dry eye	8.0	24.0	56.0
Cataract	0	20.0	40.0
Punctate keratitis	0	20.0	36.0
Pinguecula	0	0	24.0
Blepharitis	0	0	12.0
Vitreous detachment	0	8.0	8.0
Diabetic retinopathy	0	0	8.0
Corneal pigmentation	0	0	8.0
MMQS^b^			
Mean (range)	0.3 (0–1)	2.0 (2–2)	3.0 (3–3)
Schirmer test wetting length, mm			
Mean (range)	16.4 (7–35)	18.6 (7–35)	16.7 (7–40)
Ocular Symptom Questionnaire sum of worst 2 symptom scores			
Mean (range)	2.3 (0–4)	3.1 (0–4)	4.6 (4–8)

^a^ Listed ocular events are limited to those occurring in ≥2 subjects in any 1 cohort.

^b^ Study eye, investigator graded.

The overall incidence of a prior ophthalmic condition was higher in the severe MGD cohort [72% (18/25)] than in the mild/moderate MGD cohort [40% (10/25)] and non-MGD cohort [28% (7/25)] (Table 3). Dry eye was the most frequently reported prior ophthalmic condition, occurring in 8% (2/25), 24% (6/25), and 56% (14/25) of subjects in the non-MGD, mild/moderate MGD, and severe MGD cohorts, respectively.

Visual acuity testing on day 1 indicated that the great majority of study participants (61/75, 81.3%) had excellent BCVA (Snellen equivalent 20/20 or better) in both eyes, and that virtually all other participants (13/75, 17.3%) had good BCVA (Snellen equivalent 20/25 to 20/40) in the worse eye (Table 4). One study participant (1.3%) in the severe MGD cohort had a BCVA of 20/20 in the right eye and 20/250 in the left eye. Classification of study participants according to the SE of both eyes indicated that the predominant refractive types were emmetropes (23/75, 30.7%) and myopes (25/75, 33.3%), with smaller proportions of hyperopes (14/75, 18.7%) and partial emmetropes (13/75, 17.3%) (Table 4).

**Table 4.** Visual Acuity and Refractive Error of the Study Population at Study Entry

BCVA (Snellen equivalent) on day 1 in worse eye	Cohort			Overall population, (*n* = 75)
	Non-MGD, (*n* = 25)	Mild/moderate MGD, (*n* = 25)	Severe MGD, (*n* = 25)	
20/20 or better	23	23	15	61
20/25	1^a^	2^a^	5^a^	8
20/32	1^a^	0	2^b^	3
20/40	0	0	2^b^	2
20/250	0	0	1^a^	1

Refractive error on day 1^c^	Cohort			Overall population, (*n* = 75)
	Non-MGD, (*n* = 25)	Mild/moderate MGD, (*n* = 25)	Severe MGD, (*n* = 25)	
Emmetropic (both eyes)	4	11	8	23
Myopic (both eyes)	11	8	6	25
Hyperopic (both eyes)	3	4	7	14
Emmetropic/myopic (1 eye each)	7	1	3	11
Emmetropic/hyperopic (1 eye each)	0	1	1	2

^a^ BCVA in the better eye was 20/20 or better.

^b^ BCVA in the better eye was 20/32.

^c^ Refractive error defined by the SE, with emmetropic = SE of −0.5 to +0.5 diopters, myopic = SE of <−0.5 diopters, and hyperopic = SE of >+0.5 diopters.

BCVA, best corrected visual acuity; SE, spherical equivalent.

### MGD IQ findings

Comparison of MGD IQ responses across the 3 cohorts indicated, at both study visits, consistently higher item scores with increasing severity of MGD, except for item 1 (difficulty working on the computer), for which the mild/moderate and severe MGD cohorts had similar scores, and item 4 (difficulty with social activities), for which all 3 cohorts had similar scores (Table 5).

**Table 5.** Meibomian Gland Dysfunction Impact Questionnaire Item Scores (Mean, Standard Deviation) by Cohort at Day 1 and 22

Item No.		Day 1 (study entry)			Day 22 (study exit)		
		Non-MGD, (*n* = 25)	Mild/moderate MGD, (*n* = 25)	Severe MGD, (*n* = 25)	Non-MGD, (*n* = 25)	Mild/moderate MGD, (*n* = 24)	Severe MGD, (*n* = 24)
1	Difficulty working on the computer	1.3 (0.8)	1.8 (0.9) *P* = 0.031^a^	1.7 (1.5) *P* = 0.984^b^ *P* = 0.156^c^	1.2 (0.8)	1.5 (0.8) *P* = 0.033^a^	1.4 (1.3) *P* = 0.835^b^ *P* = 0.047^c^
2	Difficulty reading	1.5 (0.7)	1.8 (0.8) *P* = 0.738^a^	2.5 (1.1) *P* = 0.023^b^ *P* = 0.004^c^	1.6 (0.6)	1.8 (0.6) *P* = 0.420^a^	2.1 (1.0) *P* = 0.385^b^ *P* = 0.164^c^
3	Difficulty performing leisure activities	1.4 (0.6)	1.5 (0.7) *P* = 0.913^a^	2.2 (1.2) *P* = 0.016^b^ *P* = 0.009^c^	1.2 (0.7)	1.5 (0.8) *P* = 0.296^a^	2.0 (0.8) *P* = 0.118^b^ *P* = 0.044^c^
4	Difficulty performing social activities	1.1 (0.6)	1.1 (0.3) *P* = 0.530^a^	1.4 (1.0) *P* = 0.102^b^ *P* = 0.472^c^	1.0 (0.3)	1.1 (0.6) *P* = 0.961^a^	1.1 (0.7) *P* = 0.787^b^ *P* = 0.872^c^
5	Difficulty driving	0.6 (0.9)	1.1 (0.8) *P* = 0.276^a^	1.8 (1.1) *P* = 0.101^b^ *P* = 0.015^c^	0.6 (0.7)	1.3 (1.0) *P* = 0.594^a^	1.6 (1.0) *P* = 0.894^b^ *P* = 0.041^c^
6	Difficulty performing outdoor activities	0.7 (0.9)	0.9 (0.6) *P* = 0.564^a^	1.4 (1.0) *P* = 0.064^b^ *P* = 0.137^c^	0.9 (0.5)	1.0 (0.8) *P* = 0.473^a^	1.1 (1.0) *P* = 0.652^b^ *P* = 0.044^c^
7	Frequency of difficulty with outdoor activities	0.6 (0.7)	1.2 (0.9) *P* = 0.449^a^	1.8 (1.3) *P* = 0.112^b^ *P* = 0.007^c^	0.8 (0.6)	1.0 (0.9) *P* = 0.544^a^	1.3 (1.0) *P* = 0.238^b^ *P* = 0.004^c^
8	Time spent caring for eyes	0.3 (0.6)	0.7 (0.8) *P* = 0.260^a^	1.3 (0.9) *P* = 0.045^b^ *P* = 0.003^c^	0.3 (0.7)	0.8 (1.0) *P* = 0.047^a^	1.3 (0.5) *P* = 0.078^b^ *P* < 0.001^c^
9	Bother with time spent caring for eyes	0.1 (0.4)	0.3 (0.6) *P* = 0.104^a^	1.2 (1.0) *P* = 0.005^b^ *P* = 0.003^c^	0.3 (0.5)	0.5 (0.7) *P* = 0.434^a^	0.8 (0.9) *P* = 0.495^b^ *P* = 0.491^c^
10	Bother with appearance of eyes	0.3 (0.5)	0.6 (0.9) *P* = 0.438^a^	1.2 (1.0) *P* = 0.044^b^ *P* = 0.107^c^	0.3 (0.7)	0.8 (0.9) *P* = 0.022^a^	0.9 (1.0) *P* = 0.931^b^ *P* = 0.385^c^

Responses to items 1–7 were scored on a scale of 0 to 5. Responses to items 8–10 were scored on a scale of 0 to 4.

^a^ Mild/moderate MGD cohort versus non-MGD cohort, based on the CMH method with modified ridit scores, stratified by site.

^b^ Severe MGD cohort versus mild/moderate MGD cohort, based on the CMH method with modified ridit scores, stratified by site.

^c^ Severe MGD cohort versus non-MGD cohort, based on the CMH method with modified ridit scores, stratified by site.

CMH, Cochran–Mantel–Haenszel.

**FIG. 1.**
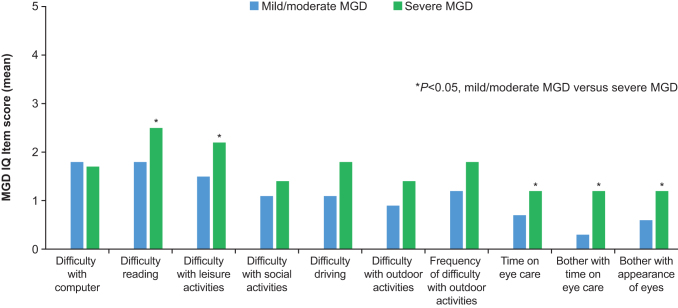
Intercohort comparison of MGD IQ item scores at day 1: mild/moderate versus severe MGD. MGD IQ, Meibomian Gland Dysfunction Impact Questionnaire.

**FIG. 2.**
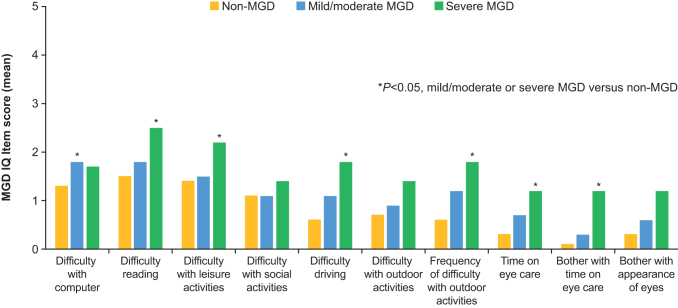
Intercohort comparisons of MGD IQ item scores at day 1: mild/moderate MGD and severe MGD versus non-MGD.

Pairwise intercohort comparisons at day 1 indicated that subjects with severe MGD experienced significantly greater difficulty with reading and performance of leisure activities, greater time spent caring for their eyes, and greater bother with eye care and the appearance of their eyes than subjects with mild/moderate MGD (all *P* < 0.05) (Fig. 1). In comparison with the non-MGD cohort, subjects with mild/moderate MGD had significantly greater difficulty working on the computer, whereas those with severe MGD had significantly greater difficulty reading, performing leisure activities, and driving; significantly more frequent difficulty with outdoor activities; spent significantly more time taking care of their eyes, and experienced significantly greater bother with eye care (all *P* < 0.05) (Fig. 2).

Corresponding comparisons at day 22 indicated that subjects with mild/moderate MGD experienced significantly greater difficulty working on the computer, more time spent on eye care, and greater bother about the appearance of their eyes than those without MGD (all *P* < 0.05). Similarly, subjects with severe MGD had significantly greater difficulty working on the computer, performing leisure and outdoor activities, and driving; experienced significantly more frequent difficulty with outdoor activities; and spent significantly more time caring for their eyes than those without MGD (all *P* < 0.05) (Table 5). In contrast to day 1 findings, no significant differences in MGD IQ item scores were noted between the mild/moderate MGD and severe MGD cohorts at day 22.

Intervisit (day 1 vs. day 22) agreement between MGD IQ responses was fair to moderate, as reflected in the proportion of subjects with the same score at both visits, which ranged from 52.1% (item 7, frequency of difficulty with outdoor activities) to 65.8% (item 4, difficulty with social activities), and in the weighted kappa statistic [range, 0.33 (item 6, difficulty with outdoor activities) to 0.58 (item 5, difficulty with driving)].

## Discussion

This analysis of patient-reported health outcome data, which were gathered prospectively in conjunction with clinical data within the clinical trial setting, indicates that the impact of MGD, as experienced by the patient, increases with the clinical severity of the condition. MGD IQ responses across the 3 cohorts translated into consistently higher mean item scores with increasing severity of MGD signs and symptoms. Although the MGD IQ instrument has yet to undergo psychometric assessment and quantitative validation, the study findings suggest that it is sensitive to the gradations of MGD severity that are encountered among the patient population. Moreover, despite the known day-to-day fluctuation in symptoms of MGD, patients' responses to the MGD IQ instrument appear to be reasonably consistent from one clinic visit to the next.

A notable finding was that the MGD IQ instrument provided a signal of greater difficulty in working on the computer among subjects with mild/moderate MGD and severe MGD compared to those without MGD. Similarly, DED has been associated with impairment of vision-related quality of life,^12,13^ including problems with reading, television, and computer use.^12^ In the decade since this study was conducted, dependence on computers and other digital display devices has grown enormously, and digital eye strain and DED symptoms have become more prevalent in the general population.^14^ Moreover, increased digital device screen exposure has been shown to be a significant risk factor for development of evaporative dry eye.^15^ Accordingly, it is likely that patients' assessments of the detrimental impact of MGD on their ability to perform computer-related activities would be even more pronounced if this study were to be repeated today.

PRO measures have gained importance in the drug development process in recent years, both for assessing therapeutic risk/benefit during regulatory review and as an efficacy endpoint in drug registration trials.^16^ In the United States, the FDA has long advocated the use of PROs as a means of incorporating the patient's voice in drug development and has implemented initiatives to foster PRO development collaboratively with sponsors.

In the absence of an MGD-specific and validated PRO instrument, clinical studies that have assessed patient-reported health outcomes in MGD have generally employed DED-specific instruments, such as the Ocular Surface Disease Index,^17–24^ the Standard Patient Evaluation of Eye Dryness (SPEED),^24–32^ the Ocular Comfort Index,^17^ the Canadian Dry Eye Assessment,^33^ the Symptom Assessment in Dry Eye,^21,24^ and the Dry Eye-Related Quality-of-Life Score (DEQS)^19^ questionnaires, which allow assessment of a range of symptoms associated with ocular discomfort. However, given that many of these symptoms are common to both MGD and DED, the available DED-specific questionnaires are unlikely to be able to differentiate between the 2 conditions.^8^

In 2011, the International Workshop on MGD brought to attention the unavailability of a MGD-specific PRO instrument and highlighted the need for research to assess the ability of specific symptom-based questionnaires to diagnose defined MGD patients and discriminate them from patients with other ocular surface problems.^34^ Similarly, a 2020 review of 24 PRO instruments used in dry eye studies noted that MGD-specific questionnaires were lacking.^35^

SPEED is a 20-item questionnaire that was developed (before issuance of the U.S. FDA's PRO Guidance in 2009) to assess severity and changes in subjective symptoms experienced over time by patients with DED.^36^ Although its psychometric properties have been only partially documented,^35^ and it lacks items on vision-related quality of life and impact on daily activities, the SPEED questionnaire has been proposed to be useful in evaluating patients with MGD.^37^ SPEED questionnaire score has been shown to correlate with clinical measures of meibomian gland function, although in patients with DED rather than MGD.^37^ The DEQS questionnaire, a 15-item instrument measuring symptom frequency and degree of disability, was developed and validated for evaluating the multifaceted effect of DED on the patient's daily activities of living.^38^ Subsequent evaluation of DEQS in patients with MGD found no significant differences in DEQS scores between those with and without MGD, which may reflect the fact that this PRO instrument was not specifically developed for use in MGD patients.^19^

Although closely related to DED, MGD is an etiologically distinct clinical entity and, as such, requires a PRO instrument that is developed and validated specifically for use in this patient population. The instrument should capture relevant and important MGD-specific symptoms and the impact of these symptoms on the patient, have good psychometric properties, and meet current FDA development standards. A reportedly “MGD-specific” symptom-based questionnaire that captures the frequency and intensity of 7 ocular symptoms (dryness, grittiness, burning, vision fluctuation, itch, soreness, and scratchiness) has recently been described.^39^ However, item selection in this case was based not on established qualitative research methodology for PRO instrument development (ie, concept elicitation and cognitive debriefing interviews conducted among the appropriate patient population), but instead on published literature reports of symptom frequency in MGD.

The MGD IQ represents the first PRO instrument developed specifically for use in the MGD patient population that complies with current FDA PRO guidance for drug registration trials. In this digital age, the ability to capture the impact of MGD on digital and other vision-related activities, as enabled by the MGD IQ, is an important feature of a quality-of-life metric that is intended for use in the clinical trial setting. It should be noted, however, that any assessment of the effect of MGD on vision-related activities is subject to potential confounding by non-MGD-related factors such as visual acuity and refractive status. Although no attempt was made to control for determinants of visual function in this observational study, the 3 study cohorts displayed generally similar distributions of visual acuities and refractive status types.

In summary, the results from this study characterize the severity of disease impact on vision-related activities and can be used to evaluate the psychometric properties of the MGD IQ to meet the FDA standards. This novel MGD IQ instrument can amplify the patient's voice on burden of disease and supports the development of patient-centric clinical research.

## Data Sharing Statement

AbbVie is committed to responsible data sharing regarding the clinical trials we sponsor. This includes access to anonymized, individual, and trial-level data (analysis data sets), as well as other information (eg, protocols, clinical study reports, or analysis plans), as long as the trials are not part of an ongoing or planned regulatory submission. This includes requests for clinical trial data for unlicensed products and indications. These clinical trial data can be requested by any qualified researchers who engage in rigorous, independent scientific research, and will be provided following review and approval of a research proposal, Statistical Analysis Plan, and execution of a Data Sharing Agreement. Data requests can be submitted at any time after approval in the United States and Europe and after acceptance of this manuscript for publication. The data will be accessible for 12 months, with possible extensions considered. For more information on the process or to submit a request, visit the following link: https://www.abbvieclinicaltrials.com/hcp/data-sharing/.html.
